# Nitric oxide in fungi: is there NO light at the end of the tunnel?

**DOI:** 10.1007/s00294-016-0574-6

**Published:** 2016-02-17

**Authors:** David Cánovas, Jose F. Marcos, Ana T. Marcos, Joseph Strauss

**Affiliations:** Department of Genetics, Faculty of Biology, University of Sevilla, Seville, Spain; Division of Microbial Genetics and Pathogen Interactions, Department of Applied Genetics and Cell Biology, University of Natural Resources and Life Sciences (BOKU), Bioresources Campus Tulln, Vienna, Austria; Department of Food Science, Institute of Agrochemistry and Food Technology (IATA), CSIC, Valencia, Spain

**Keywords:** Nitric oxide, *Aspergillus*, Fungal pathogens, Nitrate reductase, Flavohemoglobin, Development

## Abstract

Nitric oxide (NO) is a remarkable gaseous molecule with multiple and important roles in different organisms, including fungi. However, the study of the biology of NO in fungi has been hindered by the lack of a complete knowledge on the different metabolic routes that allow a proper NO balance, and the regulation of these routes. Fungi have developed NO detoxification mechanisms to combat nitrosative stress, which have been mainly characterized by their connection to pathogenesis or nitrogen metabolism. However, the progress on the studies of NO anabolic routes in fungi has been hampered by efforts to disrupt candidate genes that gave no conclusive data until recently. This review summarizes the different roles of NO in fungal biology and pathogenesis, with an emphasis on the alternatives to explain fungal NO production and the recent findings on the involvement of nitrate reductase in the synthesis of NO and its regulation during fungal development.

## Introduction

Nitric oxide (NO) is a diatomic gas, ubiquitous, globally distributed and has high reactivity with a half-live of a few seconds. In biology NO is a fascinating molecule playing multiple roles in cellular metabolism. It can be damaging through its high reactivity towards proteins causing nitrosylation, nitrosative stress and apoptosis. The “killer effect” of NO is used by higher organisms to defend against microbial pathogens (fungi and bacteria). On the other hand, NO is also beneficial as it acts as a signaling molecule, controlling essential biological processes, such as signal transduction from the cell surface into the cell, responses to abiotic and biotic stresses, as well as development in plants, or vasoconstriction and reproduction in mammals (Alderton et al. 2001; Arasimowicz-Jelonek and Floryszak-Wieczorek 2014; Gardner et al. 2010; Golombek et al. 2004; Gorren and Mayer 2007; Simontacchi et al. 2015).

NO synthesis and signaling roles have been deeply characterized in taxonomic groups as diverse as bacteria, plants and mammals. In animals, the oxidative synthesis involves the conversion of l-arginine and O_2_ into citrulline and NO by up to three isoforms of the so-called nitric oxide synthase (NOS) (Alderton et al. 2001; Gorren and Mayer 2007; Lamotte et al. 2005). In higher plants, the existence of an enzymatic l-arginine-dependent synthesis of NO has been demonstrated, although no homology with known NOS has been found in fully sequenced plant genomes (Prochazkova et al. 2014; Simontacchi et al. 2015). The reductive NO synthesis was shown to be widespread in plants, and involves the action of the nitrate reductase at saturating nitrite concentrations under reductive conditions (excess of NAPDH) (Lamotte et al. 2005; Rockel et al. 2002; Yamasaki 2000; Yamasaki and Sakihama 2000). Additional reductive pathways have been also found in mammals that allow the conversion of nitrite to NO with the involvement of different enzymes such as cytochrome* c*, P450 or deoxyhaemoglobin in the heart and wall vessels. Finally, non-enzymatic mechanisms such as conversion of nitrite to NO under acidic conditions also produce significant amounts of NO (Zweier et al. 2010).

However, and surprisingly, NO biosynthesis and function have been poorly studied in the fungal kingdom, counting over 1.5 million estimated species with a wide distribution (Stajich et al. 2009). Fungi can grow saprophytically as free organisms, engage in mutualistic interactions with other organisms or parasite plants, animals or even other fungi. Although regulation of nitrogen assimilation and metabolism has been extensively investigated in fungi (Arst and Cove 1973; Berger et al. 2008; Caddick et al. 1986; Magasanik and Kaiser 2002; Marzluf 1997; Schinko et al. 2010, 2013; Strauss et al. 1998; Todd et al. 2005), the study of NO metabolism has lagged behind despite the fact that it was shown already many years ago that fungal cells produce NO (Ninnemann and Maier 1996). However, the rather basic question of how fungi synthesize NO has remained elusive probably also because fungal genomes do not contain obvious mammalian-type NOS. It was demonstrated that fungal cells are capable of denitrification and ammonium fermentation (Takaya 2002), processes that are triggered in response to hypoxia in an attempt to survive. Under these stringent low oxygen conditions, NO is generated and activates the fungal responses to nitrosative stress (Hillmann et al. 2015). Denitrification allows fungi to respire nitrate under anoxic conditions whereas they assimilate nitrate and nitrite into amino acids under aeration. In *Fusarium* it was found that denitrification involves the action of a nitrate reductase (NR), a nitrite reductase (NiR) and a nitric oxide reductase (Nor) to transform nitrate into nitrite and further reduce it to N_2_O (Kobayashi and Shoun 1995). But how nitrite is further reduced to NO in this pathway remains obscure for the moment. Ammonium fermentation consists of the dissimilatory reduction of nitrate, in which nitrate is employed as a terminal acceptor of electrons, and is coupled to ethanol oxidation under anoxic conditions. Similarly to the assimilatory pathway, it produces ammonium, it was found to be cytosolic, and involves the assimilatory nitrate and nitrite reductases (*niaD* and *niiA*, respectively) in *A. nidulans* (Takasaki et al. 2004). Recent data from our laboratories show that full NO production requires a functional NR gene that is regulated independently from its role in nitrogen assimilation, and therefore contributes partially to NO production in *Aspergillus* (Marcos et al. 2016; Schinko et al. 2010).

## NO homeostasis in fungi

**Fig. 1:**
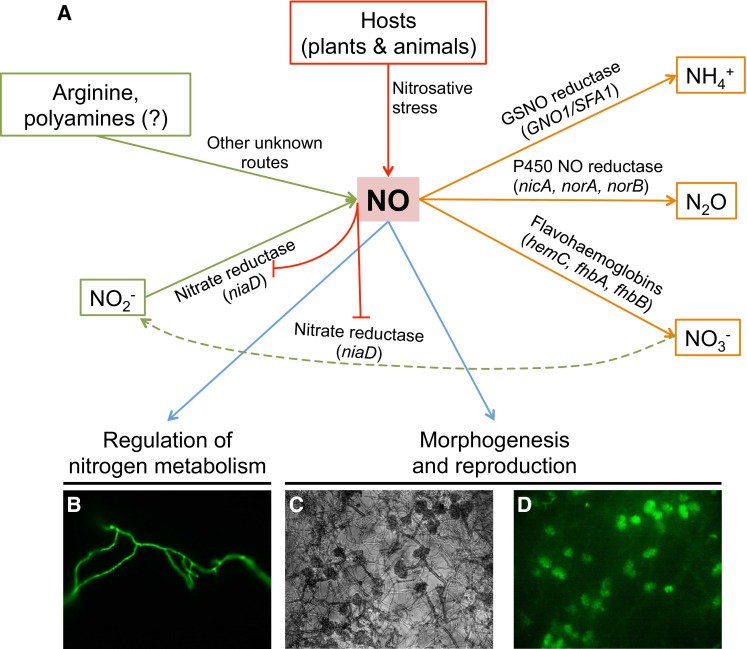
NO biosynthetic and metabolic routes known in fungi and its role in fungal biology. **a** NO in fungi can originate from biosynthetic pathways or from external sources, such as the nitrosative stress generated by animal or plant hosts to combat fungal infections. Only one reductive pathway for the synthesis of NO has been deciphered in fungi, which involves an assimilatory nitrate reductase. Four detoxification mechanisms have been identified in fungi, of which three of them result in the conversion of NO into ammonium, nitrous oxide or nitrate. The diagram includes gene names from several fungal species to provide a broad view of the NO homeostasis in the fungal kingdom. NO regulates nitrogen metabolism through the inactivation of the nitrate reductase (and maybe some other mechanisms) and it is one of the earliest signals during the transition from vegetative growth to reproduction. **b** Staining of vegetative hyphae with the NO-sensitive fluorescent dye DAF-FM diacetate. **c**, **d** Micrograph of a conidiating culture of *A. nidulans* showing strong DAF-FM diacetate signal in the conidiophores **d**

Due to the multiple roles and short life of NO, balancing the proper NO concentration at each stage of fungal life cycle is critical. This is accomplished not only through biosynthetic reactions but also by detoxifying mechanisms (Fig. 1). There was some progress through the characterization of two flavohemoglobins in *A. nidulans* by Schinko et al. (2010) who reported an essential function of these genes when the fungus grows on nitrite at low pH, which naturally releases NO through decomposition. To combat the nitrosative stress originating from developing nitrite, the authors showed that the oxidation of NO to nitrate by these flavohemoglobins is essential. The mutants generated in these studies were also useful for deciphering NO anabolism in this model fungus (see below). The role of the flavohemoglobins in NO metabolism has also been reported in other fungi including *A. oryzae* and *A. fumigatus*, yeasts, *Cryptococcus*, *Candida* and *Botrytis* (de Jesus-Berrios et al. 2003; Hromatka et al. 2005; Lapp et al. 2014; Liu et al. 2000; Philippe et al. 2003; Turrion-Gomez et al. 2010; Ullmann et al. 2004). In addition to the flavohemoglobins, other proteins have also been found to be involved in the detoxification of NO. For example, the porphobilinogen deaminase *hemC* acts by promoting the activity of the flavohemoglobins through an unknown mechanism, while the NO-inducible nitrosothionein *ntpA* scavenges NO through S-nitrosylation in *A. nidulans* (Zhou et al. 2012, 2013). A S-nitrosoglutathione (GSNO) reductase converts GSNO into ammonia and oxidized glutathione (GSSG) in *Cryptococcus neoformans* and *Magnaporthe oryzae* (de Jesus-Berrios et al. 2003; Zhang et al. 2015b).

Fungal genomes are devoid of NOS orthologs, and only distantly related NOS-like proteins could be found (Alderton et al. 2001; Gorren and Mayer 2007; Lamotte et al. 2005; Samalova et al. 2013; Zhang et al. 2015a). However, deletion of genes involved in the biosynthesis of arginine or the NOS-like gene in in the rice blast fungus *M. oryzae* (Samalova et al. 2013; Zhang et al. 2015a) and in *A. nidulans* (our own unpublished results) did not affect NO synthesis, which has hampered the progress of the studies of the fungal NO anabolic routes. Additional deletion of NR and NiR in *M. oryzae* did not impair NO synthesis either (Samalova et al. 2013; Zhang et al. 2015a). In a recent report we were able to demonstrate that fungi can synthesize NO from nitrate by means of NR (Marcos et al. 2016). To demonstrate this pathway, the combination of flavohemoglobin mutations in the same genetic background in *A. nidulans* with mutations in the nitrate assimilation pathway and its regulators was required, and allowed us to decipher the role of NR in NO biosynthesis. Surprisingly, the expression of the NR gene *niaD* and the NO levels were regulated during development even in the presence of the repressing nitrogen source ammonium (Fig. 1). The general regulator of nitrogen metabolism AreA and the nitrate pathway specific regulator NirA were responsible for this regulation (Marcos et al. 2016).

Additional tools that might help to study NO biosynthesis in fungi are provided by bioactive peptides known to increase NO production. Some of these bioactive peptides display anti-hypertensive properties and induce NO production in cultured human cells, which has been linked to their blood pressure-lowering effect (Manzanares et al. 2015). Also, the synthetic antifungal hexapeptide PAF26 specifically induces the expression of genes involved in the biosynthesis of arginine and the production of NO as part of the killing mechanism in *S. cerevisiae* (Carmona et al. 2012; Lopez-Garcia et al. 2010). Deletion of the *ARG1* gene blocks the peptide-induced NO production and increases yeast tolerance to PAF26, while the NOS inhibitor L-NAME protects yeast cells from peptide toxicity, thus supporting an arginine-derived production. Although these findings could not be translated to filamentous fungi (our own unpublished data), they indicate that bioactive peptides may add new tools to the study of NO synthesis in fungi.

## Role of NO during fungal pathogenesis

Fungi can engage in interactions with other organisms, some of which are parasitic. At this respect, it is intriguing that pathogenic fungi may employ NO as a signaling molecule to infect plants (McQuarters et al. 2014; Samalova et al. 2013), while plants seem to employ nitrosative stress (i.e. NO) to combat fungal infections. For example, *Botrytis* produces NO during infection depending on the plant host and the infection stage (Turrion-Gomez and Benito 2011) and in *M. oryzae* removal of NO resulted in decreased pathogenicity (Samalova et al. 2013). At the same time, it is well described in different plant-pathogen interactions that the infection provokes a NO burst (Delledonne et al. 1998). For example, this has been found in the case of the interaction of *Botrytis* with tobacco (Asai and Yoshioka 2009) or *Arabidopsis* (Baarlen et al. 2007). *Arabidopsis* mutant lines displaying increased NO levels (due to a mutation in the flavohemoglobin GLB1) showed increased levels of the stress-related plant hormones jasmonic acid and ethylene, and increased resistance to *Botrytis cinerea* infections, while decreased NO levels in GLB1 overexpressing lines resulted in the opposite phenotype (Mur et al. 2012). Moreover, mutants of *M. oryzae* lacking the NO-detoxifying enzyme* S*-(hydroxymethyl)-glutathione dehydrogenase (SFA1) exhibited reduced pathogenicity, and were delayed in the primary infection and growth inside the host (Zhang et al. 2015b). However, the effects of the NO burst on the plant defense to pathogens seem to be specific for each case. For example, decreased NO levels in tobacco increased the susceptibility to *Colletotrichum* but not to *Phytophtora infestans* (Asai et al. 2008). In other pathosystems deletion of the fungal flavohemoglobin did not affect *Botrytis* pathogenicity (Turrion-Gomez et al. 2010).

Supplementation of tomato fruits with arginine increased the resistance to *Botrytis* infections through NO biosynthesis in a process regulated by MAPKs (Zheng et al. 2011). The NO burst in the plant response to the pathogen infection is mediated by a cascade of MAPK and salicylic acid signaling (Asai et al. 2008; Asai and Yoshioka 2009). In this case, it was suggested that NO could be synthesized by using the nitrate-NR pathway (Asai and Yoshioka 2009; Rasul et al. 2012; Zhang et al. 2012), although intriguingly the NOS inhibitor L-NAME (an arginine analog) affected NO production (Rasul et al. 2012). The NO burst was found to occur in the adjacent areas to the infection zone. But other authors also suggest that the NO produced by the fungus during the infection diffuses into the plant (Turrion-Gomez and Benito 2011).

The scenario in the case of animal pathogens is similar to the one described in plants. For example, deletion of FHB1 and GNO1 (GSNO reductase) results in attenuated virulence of *Cryptococcus neoformans* in a murine model, whereas deletion of iNOS in the animal restored the virulence (de Jesus-Berrios et al. 2003). Similarly, YHB1 deletion in *Candida albicans* shows moderately attenuated virulence (Hromatka et al. 2005; Ullmann et al. 2004). However, the virulence defect in this case is not suppressed by deletion of the host NOS2 gene (Hromatka et al. 2005). In contrast to *Cryptococcus* and *Candida*, deletion of the flavohemoglobins and/or GSNO reductase did not impaired or attenuated *A. fumigatus* virulence (Lapp et al. 2014; Philippe et al. 2003).

## Role of NO during fungal morphogenesis and reproduction

NO has also been found to regulate morphogenesis and reproduction in several fungal species (Baidya et al. 2011; Gong et al. 2007; Hromatka et al. 2005; Maier et al. 2001; Marcos et al. 2016; Ninnemann and Maier 1996; Song et al. 2000). In this way, fungi also behave in an analogous way to mammals. *A. nidulans* poses an interesting case, since asexual and sexual reproduction (homotallic or self-mating) can occur in a single colony and are in balance depending of the external signals (Rodriguez-Romero et al. 2010). NO boosts immediately after switching from vegetative growth to the developmental programs, which suggests that it could be one of the earliest signals triggered upon induction of development, regardless of whether it is sexual or asexual. The NR gene *niaD* and the flavohemoglobin B gene *fhbB* were found to be developmentally regulated, which points to a link between metabolism and development (Marcos et al. 2016). Intriguingly, NO represses asexual reproduction while promoting the sexual one. Light, which also modulates the balance between both reproduction programs, is involved in the regulation of the expression of *fhbB* and the NO levels (Marcos et al., manuscript in preparation). Additionally, NO participates in the regulation of nitrogen metabolism during nitrate assimilation in *A. nidulans* (Marcos et al. 2016; Schinko et al. 2010, 2013). Whether these events are regulated in a similar manner or employ different mechanisms requires further studies.

## Conclusions and future perspectives

Different biosynthetic/detoxification routes exist in fungi, similarly to other organisms. It is possible that there are specific biological processes in which NO is involved that are specifically connected to different and process-specific biosynthetic routes. For example, it is not known whether the nitrate assimilation route is essential or necessary for fungal pathogenesis, either by conferring fungi the capacity to use the NO-derived nitrate as nitrogen source, or by the production of NO as a signaling molecule for other processes such as morphogenesis. It could be that the fine tuning of the NO synthesis and metabolism to maintain physiological levels is essential to maintain the proper balance to control biological processes, such as pathogenesis, growth, morphogenesis and reproduction. The extensive previous knowledge on the regulation by light and nitrogen in *Aspergillus* and the recently discovered connections to NO biology provide a useful toolbox to decipher the complex mechanism and regulation of NO biosynthesis in fungi. The regulatory pathways in which NO is involved are barely understood in fungi, and are becoming necessary to determine the complex interaction of fungal pathogens and their hosts.

## References

[CR1] Alderton WK, Cooper CE, Knowles RG (2001). Nitric oxide synthases: structure, function and inhibition. Biochem J.

[CR2] Arasimowicz-Jelonek M, Floryszak-Wieczorek J (2014). Nitric oxide: an effective weapon of the plant or the pathogen?. Mol Plant Pathol.

[CR3] Arst HN, Cove DJ (1973). Nitrogen metabolite repression in *Aspergillus nidulans*. Mol Gen Genet.

[CR4] Asai S, Yoshioka H (2009). Nitric oxide as a partner of reactive oxygen species participates in disease resistance to nectrotophic pathogen *Botryis cinerea* in *Nicotiana benthamiana*. Mol Plant-Microbe Interact MPMI.

[CR5] Asai S, Ohta K, Yoshioka H (2008). MAPK signaling regulates nitric oxide and NADPH oxidase-dependent oxidative bursts in *Nicotiana benthamiana*. Plant Cell.

[CR7] Baidya S, Cary JW, Grayburn WS, Calvo AM (2011). Role of nitric oxide and flavohemoglobin homolog genes in *Aspergillus nidulans* sexual development and mycotoxin production. Appl Environ Microbiol.

[CR8] Berger H, Basheer A, Bock S, Reyes-Dominguez Y, Dalik T, Altmann F, Strauss J (2008). Dissecting individual steps of nitrogen transcription factor cooperation in the *Aspergillus nidulans* nitrate cluster. Mol Microbiol.

[CR9] Caddick MX, Arst HN, Taylor LH, Johnson RI, Brownlee AG (1986). Cloning of the regulatory gene *areA* mediating nitrogen metabolite repression in *Aspergillus nidulans*. EMBO J.

[CR10] Carmona L, Gandia M, Lopez-Garcia B, Marcos JF (2012). Sensitivity of *Saccharomyces cerevisiae* to the cell-penetrating antifungal peptide PAF26 correlates with endogenous nitric oxide (NO) production. Biochem Biophys Res Commun.

[CR11] de Jesus-Berrios M, Liu L, Nussbaum JC, Cox GM, Stamler JS, Heitman J (2003). Enzymes that counteract nitrosative stress promote fungal virulence. Curr Biol.

[CR12] Delledonne M, Xia Y, Dixon RA, Lamb C (1998). Nitric oxide functions as a signal in plant disease resistance. Nature.

[CR13] Gardner AM, Cook MR, Gardner PR (2010). Nitric-oxide dioxygenase function of human cytoglobin with cellular reductants and in rat hepatocytes. J Biol Chem.

[CR14] Golombek DA, Agostino PV, Plano SA, Ferreyra GA (2004). Signaling in the mammalian circadian clock: the NO/cGMP pathway. Neurochem Int.

[CR15] Gong X, Fu Y, Jiang D, Li G, Yi X, Peng Y (2007). l-arginine is essential for conidiation in the filamentous fungus *Coniothyrium minitans*. Fungal Genet Biol.

[CR16] Gorren AC, Mayer B (2007). Nitric-oxide synthase: a cytochrome P450 family foster child. Biochim Biophys Acta.

[CR17] Hillmann F, Shekhova E, Kniemeyer O (2015). Insights into the cellular responses to hypoxia in filamentous fungi. Curr Genet.

[CR18] Hromatka BS, Noble SM, Johnson AD (2005). Transcriptional response of *Candida albicans* to nitric oxide and the role of the YHB1 gene in nitrosative stress and virulence. Mol Biol Cell.

[CR19] Kobayashi M, Shoun H (1995). The copper-containing dissimilatory nitrite reductase involved in the denitrifying system of the fungus *Fusarium oxysporum*. J Biol Chem.

[CR20] Lamotte O, Courtois C, Barnavon L, Pugin A, Wendehenne D (2005). Nitric oxide in plants: the biosynthesis and cell signalling properties of a fascinating molecule. Planta.

[CR21] Lapp K, Vodisch M, Kroll K, Strassburger M, Kniemeyer O, Heinekamp T, Brakhage AA (2014). Characterization of the *Aspergillus fumigatus* detoxification systems for reactive nitrogen intermediates and their impact on virulence. Front Microbiol.

[CR22] Liu L, Zeng M, Hausladen A, Heitman J, Stamler JS (2000). Protection from nitrosative stress by yeast flavohemoglobin. Proc Natl Acad Sci USA.

[CR23] Lopez-Garcia B, Gandia M, Munoz A, Carmona L, Marcos JF (2010). A genomic approach highlights common and diverse effects and determinants of susceptibility on the yeast *Saccharomyces cerevisiae* exposed to distinct antimicrobial peptides. BMC Microbiol.

[CR24] Magasanik B, Kaiser CA (2002). Nitrogen regulation in *Saccharomyces cerevisiae*. Gene.

[CR25] Maier J, Hecker R, Rockel P, Ninnemann H (2001). Role of nitric oxide synthase in the light-induced development of sporangiophores in *Phycomyces blakesleeanus*. Plant Physiol.

[CR26] Manzanares P, Salom JB, Garcia-Tejedor A, Fernandez-Musoles R, Ruiz-Gimenez P, Gimeno-Alcaniz JV (2015). Unraveling the mechanisms of action of lactoferrin-derived antihypertensive peptides: ACE inhibition and beyond. Food Funct.

[CR27] Marcos AT, Ramos MS, Marcos JF, Carmona L, Strauss J, Canovas D (2016). Nitric oxide synthesis by nitrate reductase is regulated during development in *Aspergillus*. Mol Microbiol.

[CR28] Marzluf GA (1997). Genetic regulation of nitrogen metabolism in the fungi. Microbiol Mol Biol Rev.

[CR29] McQuarters AB, Wirgau NE, Lehnert N (2014). Model complexes of key intermediates in fungal cytochrome P450 nitric oxide reductase (P450nor). Curr Opin Chem Biol.

[CR30] Mur LA, Sivakumaran A, Mandon J, Cristescu SM, Harren FJ, Hebelstrup KH (2012). Haemoglobin modulates salicylate and jasmonate/ethylene-mediated resistance mechanisms against pathogens. J Exp Bot.

[CR31] Ninnemann H, Maier J (1996). Indications for the occurrence of nitric oxide synthases in fungi and plants and the involvement in photoconidiation of *Neurospora crassa*. Photochem Photobiol.

[CR32] Philippe B, Ibrahim-Granet O, Prevost MC, Gougerot-Pocidalo MA, Sanchez Perez M, Van der Meeren A, Latge JP (2003). Killing of *Aspergillus fumigatus* by alveolar macrophages is mediated by reactive oxidant intermediates. Infect Immun.

[CR33] Prochazkova D, Haisel D, Pavlikova D (2014). Nitric oxide biosynthesis in plants—the short overview. Plant Soil Environ.

[CR34] Rasul S, Dubreuil-Maurizi C, Lamotte O, Koen E, Poinssot B, Alcaraz G, Wendehenne D, Jeandroz S (2012). Nitric oxide production mediates oligogalacturonide-triggered immunity and resistance to *Botrytis cinerea* in *Arabidopsis thaliana*. Plant Cell Environ.

[CR35] Rockel P, Strube F, Rockel A, Wildt J, Kaiser WM (2002). Regulation of nitric oxide (NO) production by plant nitrate reductase in vivo and in vitro. J Exp Bot.

[CR36] Rodriguez-Romero J, Hedtke M, Kastner C, Muller S, Fischer R (2010). Fungi, hidden in soil or up in the air: light makes a difference. Annu Rev Microbiol.

[CR37] Samalova M, Johnson J, Illes M, Kelly S, Fricker M, Gurr S (2013). Nitric oxide generated by the rice blast fungus *Magnaporthe oryzae* drives plant infection. New Phytol.

[CR38] Schinko T, Berger H, Lee W, Gallmetzer A, Pirker K, Pachlinger R, Buchner I, Reichenauer T, Guldener U, Strauss J (2010). Transcriptome analysis of nitrate assimilation in *Aspergillus nidulans* reveals connections to nitric oxide metabolism. Mol Microbiol.

[CR39] Schinko T, Gallmetzer A, Amillis S, Strauss J (2013). Pseudo-constitutivity of nitrate-responsive genes in nitrate reductase mutants. Fungal Genet Biol.

[CR40] Simontacchi M, Galatro A, Ramos-Artuso F, Santa-Maria GE (2015). Plant survival in a changing environment: the role of nitric oxide in plant responses to abiotic stress. Front Plant Sci.

[CR41] Song N-K, Jeong C-S, Choi H-S (2000). Identification of nitric oxide synthase in *Flammulina velutipes*. Mycologia.

[CR42] Stajich JE, Berbee ML, Blackwell M, Hibbett DS, James TY, Spatafora JW, Taylor JW (2009). The fungi. Curr Biol.

[CR43] Strauss J, Muro-Pastor MI, Scazzocchio C (1998). The regulator of nitrate assimilation in ascomycetes is a dimer which binds a nonrepeated, asymmetrical sequence. Mol Cell Biol.

[CR44] Takasaki K, Shoun H, Yamaguchi M, Takeo K, Nakamura A, Hoshino T, Takaya N (2004). Fungal ammonia fermentation, a novel metabolic mechanism that couples the dissimilatory and assimilatory pathways of both nitrate and ethanol. Role of acetyl CoA synthetase in anaerobic ATP synthesis. J Biol Chem.

[CR45] Takaya N (2002). Dissimilatory nitrate reduction metabolisms and their control in fungi. J Biosci Bioeng.

[CR46] Todd RB, Fraser JA, Wong KH, Davis MA, Hynes MJ (2005). Nuclear accumulation of the GATA factor AreA in response to complete nitrogen starvation by regulation of nuclear export. Eukaryot Cell.

[CR47] Turrion-Gomez JL, Benito EP (2011). Flux of nitric oxide between the necrotrophic pathogen *Botrytis cinerea* and the host plant. Mol Plant Pathol.

[CR48] Turrion-Gomez JL, Eslava AP, Benito EP (2010). The flavohemoglobin BCFHG1 is the main NO detoxification system and confers protection against nitrosative conditions but is not a virulence factor in the fungal necrotroph *Botrytis cinerea*. Fungal Genet Biol.

[CR49] Ullmann BD, Myers H, Chiranand W, Lazzell AL, Zhao Q, Vega LA, Lopez-Ribot JL, Gardner PR, Gustin MC (2004). Inducible defense mechanism against nitric oxide in *Candida albicans*. Eukaryot Cell.

[CR6] Van Baarlen P, Woltering EJ, Staats M, van Kan JA (2007). Histochemical and genetic analysis of host and non-host interactions of *Arabidopsis* with three *Botrytis* species: an important role for cell death control. Mol Plant Pathol.

[CR50] Yamasaki H (2000). Nitrite-dependent nitric oxide production pathway: implications for involvement of active nitrogen species in photoinhibition in vivo. Philos Trans R Soc Lond B Biol Sci.

[CR51] Yamasaki H, Sakihama Y (2000). Simultaneous production of nitric oxide and peroxynitrite by plant nitrate reductase: in vitro evidence for the NR-dependent formation of active nitrogen species. FEBS Lett.

[CR52] Zhang H, Li D, Wang M, Liu J, Teng W, Cheng B, Huang Q, Wang M, Song W, Dong S, Zheng X, Zhang Z (2012). The *Nicotiana benthamiana* mitogen-activated protein kinase cascade and WRKY transcription factor participate in Nep1(Mo)-triggered plant responses. Mol Plant-Microbe Int MPMI.

[CR53] Zhang Y, Shi H, Liang S, Ning G, Xu N, Lu J, Liu X, Lin F (2015). MoARG1, MoARG5,6 and MoARG7 involved in arginine biosynthesis are essential for growth, conidiogenesis, sexual reproduction, and pathogenicity in *Magnaporthe oryzae*. Microbiol Res.

[CR54] Zhang Z, Wang J, Chai R, Qiu H, Jiang H, Mao X, Wang Y, Liu F, Sun G (2015). An S-(hydroxymethyl)glutathione dehydrogenase is involved in conidiation and full virulence in the rice blast fungus *Magnaporthe oryzae*. PLoS One.

[CR55] Zheng Y, Sheng J, Zhao R, Zhang J, Lv S, Liu L, Shen L (2011). Preharvest L-arginine treatment induced postharvest disease resistance to *Botrysis cinerea* in tomato fruits. J Agric Food Chem.

[CR56] Zhou S, Narukami T, Nameki M, Ozawa T, Kamimura Y, Hoshino T, Takaya N (2012). Heme-biosynthetic porphobilinogen deaminase protects *Aspergillus nidulans* from nitrosative stress. Appl Environ Microbiol.

[CR57] Zhou S, Narukami T, Masuo S, Shimizu M, Fujita T, Doi Y, Kamimura Y, Takaya N (2013). NO-inducible nitrosothionein mediates NO removal in tandem with thioredoxin. Nat Chem Biol.

[CR58] Zweier JL, Li H, Samouilov A, Liu X (2010). Mechanisms of nitrite reduction to nitric oxide in the heart and vessel wall. Nitric Oxide Biol Chem.

